# Letter from Paris

**Published:** 1867-09

**Authors:** J. D'Oyley-Evans


### CORRESPONDENCE.

### *Letter from Paris.*

PARIS, *July* 10th, 1867.

MESSRS. EDITORS:—

I have been so occupied since my return, that I have had little leisure to make observations upon new discoveries in medicine, or inventions in surgery. I must therefore be quite brief in what I have to say this month.

I learn that Dr. Velpeau has presented to the Academy of Science a curious paper by Dr. Rainbert on a new method of introducing medicines into the aninal economy; viz. by the nostrils. He has experimented with morphia, which, he claims, introduced in that way, will cure the most violent headache. I shall endeavor, by the time I send off my next letter, to ascertain some definite facts in connection with the new method, and if interesting or useful, will give you some detail of them.

At present I shall confine myself to some observations on the effect of chlorate of potash upon the gums and mouth of patients, suffering from suppuration, and salivation consequent upon the ingestion of mercury.

In 1856 Dr. Ricord, and subsequently Dr. Fournier, his pupil, published some observations on a new property, which they had discovered in the chlorate of potash, viz. that of neutralizing the effects of mercury on the animal economy. The *Abeille medicale* now gives an account of certain observations made by Dr. Laborde on the subject, at the Hospital de la Charitè.

It is well known that when mercury is taken in strong doses, or without interruption for a certain number of days, it produces salivation, and sometimes inflammation of the stomach. But if at the same time chlorate of potash be administered under the form of a potion, and in some cases also by friction, these effects will be prevented, or, if they already exist, cured in a few days. Dr Laborde's experiments form two series. In the first, inflammation of the stomach, and salivation having been produced by the use of mercury, the latter was suspended, and chlorate of potash, at the rate of four or five grammes* per day dissolved in water, and sweetened with julep was administered, when the cure was effected in the course of a few days.

In the second series of experiments, the use of mercury was *not* suspended, but the chlorate of potash administered at the same time, and with the same success.

If the chlorate was suspended, the mercury symptoms would return immediately, to disappear again as soon as chlorate was administered. If the proto-iodide of mercury for example, be administered conjointly with the chlorate of potash, it may be continued for the space of two months, in doses of from 15 to 20 centigrammes per diem, without producing any effect whatever on the gums ; but no sooner is the chlorate suspended than the mecurial symptoms appear. Except in cases of great intensity, the cure of the latter is effected in about four days.

Dr. Laborde also confirms Dr. Ricord's statement, that the administration of chlorate of potash does not, in the slightest degree diminish the curative properties of mercury.

J. D'Oyley-Evans, D.D.S.

---

* A grammes is 15.43 grains

